# Auditory time perception impairment in children with developmental dyscalculia

**DOI:** 10.1016/j.ridd.2024.104733

**Published:** 2024-06

**Authors:** Elisa Castaldi, Francesca Tinelli, Gasperini Filippo, Mariaelisa Bartoli, Giovanni Anobile

**Affiliations:** a Department of Neuroscience, Psychology, Pharmacology, and Child Health, University of Florence, Florence, Italy; b Department of Developmental Neuroscience, IRCCS Fondazione Stella Maris, Pisa, Italy

**Keywords:** Developmental dyscalculia, Auditory time perception, ATOM, Magnitude processing

## Abstract

Developmental dyscalculia (DD) is a specific learning disability which prevents children from acquiring adequate numerical and arithmetical competences. We investigated whether difficulties in children with DD spread beyond the numerical domain and impact also their ability to perceive time. A group of 37 children/adolescent with and without DD were tested with an auditory categorization task measuring time perception thresholds in the sub-second (0.25–1 s) and supra-second (0.75–3 s) ranges. Results showed that auditory time perception was strongly impaired in children with DD at both time scales. The impairment remained even when age, non-verbal reasoning, and gender were regressed out. Overall, our results show that the difficulties of DD can affect magnitudes other than numerical and contribute to the increasing evidence that frames dyscalculia as a disorder affecting multiple neurocognitive and perceptual systems.

## Highlights

• People with developmental dyscalculia (DD) struggle with time management.
• We studied auditory time perception in children with dyscalculia (DD) and controls.
• Auditory time thresholds of sub- and supra-second stimuli were impaired in DD.
• DD is a multicomponential disorder that can affect magnitudes other than numbers.

What this paper adds

In addition to difficulties in learning mathematics and dealing with numerical information, individuals with developmental dyscalculia often fail in organizing daily commitments based on activity durations. This anecdotical evidence begs the question of whether basic time perception abilities, such as the ability to perceive duration of simple sounds or images, might also be impaired in this disorder. Up until today, only a small number of studies have investigated time perception in relation to dyscalculia and come to contradictory conclusions. The inconsistency of results might be related to the complexity and heterogeneity of tasks used. Strikingly, all previous studies have investigated time perception in relation to dyscalculia by using visual stimulation, a modality which is known to be less reliable compared to the auditory one for time perception. No experiment so far has tested this population with purely auditory stimuli. This would be a more stringent test as duration judgments are more precise when intervals are delimited by auditory compared to visual markers. In this study, for the first time, we psychophysically measured auditory time thresholds in age-matched children with and without dyscalculia using a simple and fast (4-minutes) categorization task of a single auditory stimulus. This task is easier than other tasks, making it suitable for testing children. We found clear evidence for time perception impairments in children with dyscalculia at two temporal ranges (sub- and supra-second ranges) thought to rely on partially segregated neural mechanisms. These results frame dyscalculia as a multi-componential disorder affecting magnitudes other than the numerical.

## 1 Introduction

Time perception is critical in several aspects of our daily life from recreational activities, such as dancing or catching a ball, to determining the successful integration of the individual in the society, for example being able to reach the workplace on time. Learning and manipulating temporal units of measurements is also an essential part of the primary school curriculum which requires children to learn both theoretical notions (e.g. how many seconds are in a minute) as well as solving problems on measurement of time. Interestingly, there is evidence that timing abilities are predictive of mathematical competences in primary school children (Kramer et al., 2011, Odic et al., 2016, Skagerlund and Träff, 2016, Hamamouche and Cordes, 2019), even when controlling for age (Hamamouche & Cordes, 2019) and domain-general abilities, such as working memory or non-mathematical reasoning (Kramer et al., 2011, Odic et al., 2016). Although the reason for this correlation is still a matter for debate, one possibility is that formal math and time discrimination share the ability to order, process and manipulate magnitudes in general, regardless of their being temporal or numerical (Skagerlund & Träff, 2016). At the lower end of the variation in mathematical abilities, children with developmental dyscalculia (DD) may encounter difficulties learning number facts, arithmetical procedures (Geary, 1993, Butterworth, 2005) and even mastering the most basic numerical abilities, such as counting (Geary et al., 1992, Geary et al., 2007, Wilson et al., 2006) or numerically comparing sets of objects (Piazza et al., 2010, Mazzocco et al., 2011, Anobile et al., 2018). These difficulties can persist into adulthood and despite life-long math training (Mejias et al., 2012, Wilson et al., 2015, Castaldi et al., 2020, Castaldi et al., 2020, Kaufmann et al., 2020). Interestingly, in addition to the pervasive impairment when dealing with numerical information, individuals with DD often complain about their difficulty in organizing daily commitments based on activity durations (Cappelletti et al., 2011). While these anecdotal reports can simply arise from a more general calculation impairment applied to the temporal domain, some studies investigated whether they might reflect a more basic impairment in sensing time. Studies on the neurotypical population suggested that time perception might rely on multiple “clocks” which operate over multiple time scales rather than by a unitary mechanism (Buhusi & Meck, 2005). In particular, perception of sub- and supra-second durations are assumed to be dependent on distinct neural substrates and cognitive strategies. Short intervals of up to one second are thought to be processed automatically by a subcortical network linked to motor control, while perception of longer supra-second intervals might be more cognitive-mediated and involve cortical areas like the frontal and parietal cortices (Lewis and Miall, 2003b, Wiener et al., 2010).

Previous research exploring time perception in individuals with DD yielded varied conclusions regarding the presence of time perception impairments in this population, as well as the specific time ranges affected when impairments were observed. Cappelletti et al. (2011) asked adult participants to judge which of two sequentially presented bars was displayed for the longer duration and found that time discrimination thresholds in the sub-second range (360–840 ms) were not affected in individuals with DD if numbers were not presented during the trial. Time discrimination thresholds were however disrupted when the bars were preceded by numbers, even if these were irrelevant to the task, and concluded that time perception was spared in individuals with DD, although the difficulties with numerical stimuli can interfere with time perception. In contrast to the evidence of spared sub-second time processing abilities in adults with DD reported by Cappelletti et al. (2011), two studies found that, even when numbers were not part of a task, 10–12 year old children with DD committed more errors (higher percent incorrect responses) when asked to establish which of two circles lasted longer and underestimated durations more frequently than age-matched children without DD (Vicario et al., 2012 tested durations: 310–500 ms; Pellerone, 2013 tested durations: 300–500 ms). Results in literature are inconsistent even when investigating longer supra-second durations in individuals with DD. Some studies found higher duration discrimination thresholds in 10-year-old children with DD (Skagerlund & Träff, 2014) and adults (Gilaie-Dotan et al., 2014) with respect to their age-matched peers without DD when comparing supra-second durations of visual stimuli (Gilaie-Dotan et al., 2014 durations tested: around 12 s; Skagerlund & Träff, 2014 duration tested: 1500–6000 ms), and these difficulties could not be attributed to differences in non-verbal IQ or working memory capacity (Skagerlund & Träff, 2014). On the contrary, other studies using similar tasks and durations found that the percentage of incorrect responses when comparing supra-second durations was comparable in 8–9 year old children with and without DD (Vicario et al., 2012 tested durations: 1280–1520 m; Pellerone, 2013 tested durations: 1200–1500 ms). Hurks et al. (2014) calculated the ratio of the children’s reproduced (subjective) duration to the presented (objective) duration and found that 10–14 year old children with DD were less accurate when they had to translate subjectively experienced supra-second durations (3–45 s) into verbally stated units or vice versa, (e.g. when having to verbally estimate how long an interval lasted or to produce an interval for a numerically determined duration), but not when they had to reproduce durations (time reproduction task). Comparable reproduction errors of supra-second intervals between 8-year-old children with and without DD were also found by Vicario tested durations:, ms) et al., (1500–1900). However, another study found that, even when tested with a reproduction task, and using partially comparable durations as Hurks et al. (2014), 9-year-old children with DD made more errors and were less precise than the control group when reproducing long (4–14 s) intervals (Cester et al., 2017).

Overall, these studies suggest that time perception may be impaired in individuals with DD, however the heterogeneity of the results call for further investigation.

The finding that dyscalculia may (or may not) negatively impact perception of continuous dimensions other than number is often taken as evidence in support of (or against) the existence of a shared system for magnitudes processing as proposed by the ATOM theory (Walsh, 2003), according to which a common and general system is recruited for processing space, time and number in individuals considered to be neurotypical. Support for this theory comes from evidence of behavioral interference effects, such as perceptual biases occurring across magnitudes (e.g. more numerous stimuli are perceived as lasting longer compared to less numerous stimuli and vice versa, Javadi & Aichelburg, 2012; Togoli et al., 2021) or across-magnitudes adaptation effects (e.g. Tsouli et al., 2019). Moreover, the ATOM theory is supported by neuroimaging studies showing that the parietal cortex is recruited during a variety of space, time and number tasks (Bueti & Walsh, 2009). Interestingly, there is evidence that continuous magnitudes are represented in the parietal cortex irrespective of the sensory modality. For example, the parietal cortex was activated when counting or judging the approximate number of both visual and auditory events (sequences of flashes or tones Piazza et al., 2006). Activation of this region was also reported for duration estimation of visual stimuli for both sub- and supra- second intervals (Lewis & Miall, 2003a) and disruption of the functionality of this area by means of repetitive transcranial magnetic stimulation interfered with duration judgments of both visual and auditory stimuli (Bueti et al., 2008). Importantly, the parietal cortex is activated during arithmetic calculation (Simon et al., 2002, Pinel et al., 2007, Pinel and Dehaene, 2010, Castaldi et al., 2020, Arsalidou et al., 2018, Arsalidou and Taylor, 2011) and functional and anatomical abnormalities of this region have been reported in children and adults with DD (Kaufmann et al., 2011, Iuculano, 2016, Castaldi et al., 2020), probably constituting the neurobiological origin of their numerical and arithmetical impairments. Given the multimodal nature of the parietal cortex, and in the light of the ATOM theory, it can be expected that impairment at this level might also generate difficulties with magnitudes processing (in general) and in sensory modalities other than the visual one.

Surprisingly, all the above reviewed studies investigated time perception in individuals with DD using visual stimuli, and no experiment so far has tested this clinical population with purely auditory stimuli. Duration discrimination thresholds are lower for auditory compared to visual stimuli in individuals considered to be neurotypical (Grondin, 1993, Merchant et al., 2008), therefore judging temporal intervals is more difficult when these are delimited by visual compared to auditory markers. Using an easier task, by testing time perception using the auditory modality might therefore be a more stringent test for determining the presence of time perception impairments in children with DD. Moreover, testing time perception of both sub- and supra- seconds within the same sample of participants might help to determine whether one or multiple time ranges are affected, thus providing a more comprehensive characterization of time perception in children with DD. To these aims, in the present study we psychophysically measured auditory time thresholds in children with DD and neurotypical age-matched controls in both the sub- and supra- second ranges using a categorization task of a single stimulus. This task is fast (4-minutes) and simpler than the discrimination, reproduction or other tasks previously used in literature, making it suitable for testing clinical populations and children. In the light of the results from previous studies on individuals with DD, as well as of the prediction of the ATOM theory, we expect lower sensory precision in children with DD in at least one of the duration ranges tested.

## 2 Materials and methods

### 2.1 Power analysis

A priori power analysis was performed using G*Power software (version 3.1) to determine the required sample size needed to reliably detect differences in time perception sensory precision between two groups. The effect size was estimated from Skagerlund et al. (2014) and Gilaie-Dotan et al. (2014) as these are the two more recent studies reporting sensory thresholds in participants with and without DD. We performed the analysis with an ⍺ = 0.05 and a power of 0.85 and found that the required sample size was 13 or 15 participants depending on whether the analysis was performed using the results by Skagerlund et al. (2014) on children or by Gilaie-Dotan et al. (2014) on adults respectively.

### 2.2 Participants

37 children participated in the study. The sample included 22 children without DD (12 females, mean age = 11 years, range 8–16) and 15 children with DD (8 females, mean age = 10 years, range 8–15). All participants were Italian, and their native language was Italian. The experiment comprised two sessions investigating time perception in the millisecond and second ranges respectively; however two participants, one in the DD group and one in the control group, were not available to complete both sessions and were tested only with stimuli varying within the sub-second range. Participants with DD were enrolled among those referred to the Stella Maris Foundation Institute in Pisa, one of the main centers for neurodevelopmental disorders in Italy. Diagnosis of DD was made by a team of specialists after a thorough clinical assessment. Specifically, neuropsychiatrists evaluated children for the presence of neurological/psychiatric disorders and reviewed their medical history, while neuropsychologists and speech therapists detailed their neuropsychological profile based on standardized tests. In line with the DSM-5, children received diagnosis of DD if they complied with the following criteria: (1) having no neurological or sensory impairments, no psychiatric comorbidities, no current or past pharmacological treatment, (2) scoring at least 80 on the total intelligence quotient (TIQ) of the Wechsler Intelligence Scale for Children-IV (WISC-IV), (3) demonstrating significant impairments on the Italian battery for developmental dyscalculia (BDE2, a standardized test evaluating a wide range of mathematical abilities). BDE2 included multiple subtests evaluating the children’s ability to count (from 80 to 140 and back), write and read three-to-six-digit numbers, perform mental arithmetic (two tests, one testing tables), establish which out of three numbers is the largest and to locate a number along a number line. Scores in these tests were combined into a single math-index score. The control group included neurotypical children with no medical history, no learning disabilities, and typical mathematical abilities for their age (as measured by the BDE2). The two groups were matched by age and non-verbal IQ, as measured by the Raven Colored Progressive Matrix-CPM or Progressive Matrix-PM, depending on chronological age, in controls and by the Visual Perceptual Reasoning Score (IRP) index from WISC-IV in the group of children with dyscalculia. Reading abilities were also screened in a subgroup of participants (9 children without DD and 11 children with DD) by asking them to read lists of words and non-words as well as a short passage of text. Reading abilities were within the neurotypical range for all control participants, whereas 8 children with DD presented reading difficulties of clinical relevance.

The study was approved by the Ethics Committee of the Meyer’s Hospital (n. 248/2020 ID-DNATN "Attention, Time and Numeracy in children and adolescents with neurodevelopmental disorders"). Informed parental consent was obtained for each participant before the study. All experiments were performed in accordance with relevant guidelines and regulation.

### 2.3 Time perception

The paradigm used to measure time sensory thresholds was the same as the one used in a previous study investigating time perception in individuals with ADHD (Anobile et al., 2022). The task required participants to categorize sound durations. Before the testing phase, participants listened to four sounds, two with “long” and two with “short” durations and they were told that these corresponded to the extreme durations (no responses were required in this phase). After this initial “anchoring” phase, a single sound (500 Hz, 80 dB pure tone) was played, and participants were asked to categorize it as “short” or “long”. Two different timing ranges were tested in separate sessions. Sounds played to measure sensory thresholds within the sub-second range varied from 0.25 ms to 1 s (geometric mean of 0.5 ms), while those played to measure thresholds within the second range varied from 0.75 ms to 3 s (geometric mean of 1.5 ms). Each time range was sampled into 11 equal steps spanning 1 octave around its geometric mean. As a result, sounds within the sub-second range lasted 0.25, 0.28, 0.33, 0.38, 0.43, 0.5, 0.57, 0.66, 0.75, 0.87, 1 s, while those in the second range lasted 0.75, 0.86, 1, 1.13, 1.3, 1.5, 1.72, 1.98, 2.27, 2.61, 3 s. Sound duration was randomly selected at every trial and each duration was tested 4 times within a single session. In total participants were tested with 44 trials for each range. Participants spelled out the category to which they thought the sound belong to (either “long” or “short”) and the researcher entered the response by keypress. The proportion of “long” responses were plotted against the stimuli duration (log scale) and fitted with a cumulative Gaussian error function. The point of subjective equality (PSE) was defined as the duration corresponding to the 50% point of the fit (accuracy). The just notable difference (JND) was defined as the difference between 50% and 75% and was used to estimate Weber Fraction (10^JND-1), and index of sensory precision.

### 2.4 Data analysis

To compare the measurements of non-verbal reasoning that were collected using two different tests (the Visual Perceptual Reasoning Score (IRP) measured by WISC-IV for children with DD and the Raven matrices for children without DD) we converted both these indices into z-scores, according to the normative age-standardized data provided by the test manuals. The same was done for the word and non-word standardized reading tests which were then averaged to provide an index of reading abilities. T-tests were used to compare age, non-verbal IQ, mathematical and reading performance between the two groups.

As the dataset contained unequal sample sizes (22 controls and 15 participants with DD) time sensory thresholds (Weber fractions) and accuracy (PSEs) were compared across groups by means of a linear mixed model. PSEs or Wfs were entered as the dependent variable, while group (controls and participants with DD) and duration range (milliseconds, seconds) were entered as fixed effects. Participants were entered as random effect. For all conditions PSEs and Wfs were normally distributed (Shapiro-Wilk, all p > 0.05). Base ten logarithms of Bayes Factors (Log10 Bayes Factors, LBF) are also reported alongside frequentist statistics. By convention, LBF > 0.5 is considered to lend substantial evidence in favor of a difference between groups (the alternative hypothesis) and LBF < − 0.5 substantial evidence in favor of no difference between groups (the null hypothesis). Absolute values greater than 1 and 2 are considered strong and definitive evidence respectively.

## 3 Results

### 3.1 Demographical and neuropsychological data

The DD and control groups were matched for age (t(35) = 0.65, p = 0.52, LBF = − 0.42) and non-verbal IQ (t(33) = − 1.2, p = 0.23, LBF = − 0.24, see Table 1). The two groups differed in mathematical abilities (t(35) = 10.9, p < 0.001, LBF = 9.7), with the DD group performing on average more than 2 standard deviations below the normative mean reported for children of the same age by the BDE2 scoring manual. Analysis of the subgroups for which reading scores were available showed that they also differed for reading abilities (t(18) = 4.1, p < 0.001, LBF = 1.6).

**Table 1:** Descriptive characteristics of the individuals with DD. TIQ = Total Intelligence Quotient from WISC-IV, IRP = z-score Visual Perceptual Reasoning from WISC-IV; Math = composite index from BDE2; Reading = average z-scores from word and non-word reading tests. n.a.= not available.

Participants	Gender	TIQ	IRP	Math	Reading
1	F	98	1.27	-2.55	-9.19
2	M	94	0.73	-2.74	-5.84
3	F	82	0.40	-2.61	-1.17
4	F	95	1.00	-2.59	n.a.
5	F	96	0.53	-1.55	-5.73
6	M	97	0.87	-2.07	-5.02
7	M	106	1.27	-2.94	-1.71
8	M	87	0.00	-2.13	n.a.
9	F	94	0.53	-3.71	-1.69
10	M	90	-0.07	-1.66	n.a.
11	M	102	1.00	-3.42	-3.45
12	M	98	0.13	-1.27	n.a.
13	F	114	2.00	-1.59	-1.73
14	F	109	0.87	-1.63	-0.9
15	F	83	-0.13	-2.56	-0.93

### 3.2 Time perception sensory precision

**Fig. 1:**
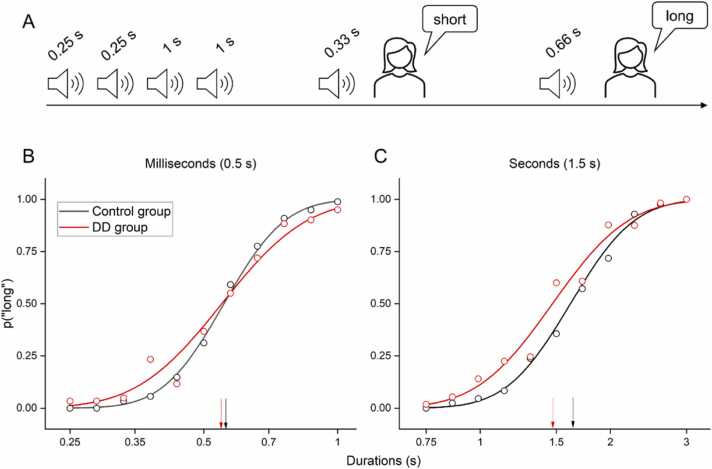
Paradigm and aggregate results. (A) At the beginning of the trial four “anchoring” sounds were played, defining the shortest (0.25 s or 0.75 s for the sub-second and second conditions respectively) and the longest sounds (1 s or 3 s for the sub-second and second conditions respectively) that demarcated the range extremes. Participants were asked to categorize the following sounds as being “short or long”. (B, C) Aggregate psychometric functions for DD (red lines and symbols) and control groups (black lines and symbols) obtained when measuring time perception in the sub-second (B) or second ranges. Arrows identify the PSEs.

All participants were able to perform the auditory time categorization task (Fig. 1A), however with different precision across groups. The psychometric curves obtained by aggregating data across participants while they categorized sounds in the millisecond and second ranges are shown in Fig. 1B and C respectively. In both time ranges the psychometric functions were steeper in the control (black line) than in the DD (red line) group, indicating lower precision in the group with DD.

**Fig. 2:**
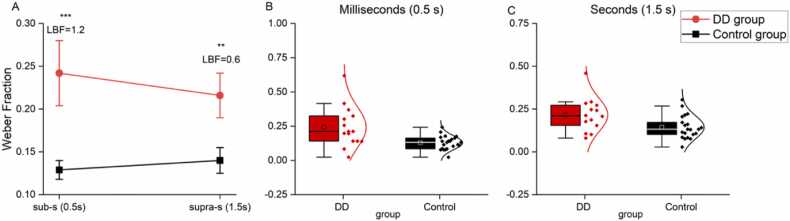
Time perception thresholds. (A) Between participants’ average time perception thresholds in the sub-second and second ranges divided by groups (DD group, red; controls, black). Error bars are standard errors of the mean; * **p < 0.005, * *p < 0.01. (B–C) Individual time thresholds distributions for the two groups and timing conditions. Filled diamonds represents individual subjects, open squares report the mean.

A similar pattern of results was obtained by fitting the psychometric curves on the data provided by each participant. Average sensory precision thresholds (Weber fractions) for each group and time range are reported in Table 2 and showed in Fig. 2A, while Fig. 2B and C display individual time thresholds. On average, sensory thresholds were higher (indicating lower precision) in the group with DD compared to the control group in both time ranges (for the millisecond range: DD group: 0.24 ± 0.15 vs control group: 0.13 ± 0.05, t(35) = 3.3, p = 0.002, d = 1.1, LBF = 1.2; for the second range: DD group: 0.22 ± 0.1 vs control group: 0.14 ± 0.07, t(33) = 2.6, p = 0.01, d = 0.9, LBF = 0.6). We entered the Weber fractions in a linear mixed model with time range (milliseconds, seconds) and group (DD, controls) as fixed factors and participants as random effect. The main effect of group was significant (F(1,35) = 14.17, p < 0.001), while the interaction between time range and group was not (F(1,35) = 1.13, p = 0.29), suggesting that the sensory precision impairment was present in both time ranges in the group with DD. Moreover, the main effect of time range was not significant (F(1,35) = 0.09, p = 0.76), suggesting that thresholds were overall similar across tasks.

**Table 2:** Time thresholds (Weber Fraction) in the two groups. Two tailed t-tests, α Bonferroni corrected 0.05/2 = 0.025.

Tasks	Group	Mean (SD)	p-value	Cohen’s d	LBF
Time millisecond range (0.5 s)	Control	0.13 (0.05)	0.002	1.1	1.2
	DD	0.24 (0.15)			
Time second range (1.5 s)	Control	0.14 (0.07)	0.01	0.8	0.6
	DD	0.22 (0.1)

To explore the specificity of the impairment found in the group with DD we performed the linear mixed model with time thresholds as the dependent variable, time range (milliseconds, seconds) and groups (DD, controls) as fixed factors, participants as random effects and age, non-verbal reasoning, and gender as covariates. Even when partialling out the effect of these covariates, the result remained unchanged with a significant effect of the group (F(1,33) = 11.6, p = 0.002), no effect of time range (F(1,33) = 0.11, p = 0.74) nor interaction (F(1,33) = 0.97, p = 0.33).

### 3.3 Time perception accuracy

**Fig. 3:**
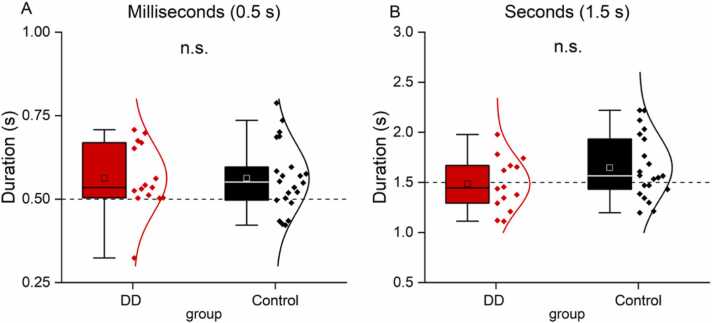
Time perception accuracy. Point of subjective equality (PSEs) in the sub-second (A) and second (B) ranges across participants of the DD (red) or control (black) group. The physical value of the reference stimuli (0.5 and 1.5 s) is marked by dotted lines. n.s. not statistically different.

After having established that time perception precision was lower in the group with DD, we tested whether the time perception accuracy was also biased compared to the veridical by analyzing differences in the PSE (Point of Subjective Equality, see Table 3 and Fig. 3). On average PSEs were very similar across groups in both time ranges (for the millisecond range: DD group: 0.56 ± 0.1 vs control group: 0.56 ± 0.1, t(35) = −0.008, p = 0.9, LBF = −0.5; for the second range: DD group: 1.6 ± 0.3 vs control group: 1.5 ± 0.3, t(33) = 1.57, p = 0.1, LBF = −0.07). We entered these values in a linear mixed model with time range (milliseconds, seconds) and group (DD, controls) as fixed factors and participants as random factor. There was no main effect of group (F(1,35) = 2.03, p = 0.16), nor a significant interaction between group and time range (F(1,35) = 3.01, p = 0.09), suggesting that judgments were comparable between DD and control groups both when they categorized stimulus duration in the millisecond (Fig. 3A) as well as in the second (Fig. 3B) ranges.

**Table 3:** Descriptive statistics. Points of subjective equality (PSEs) in the two groups.

Tasks	Group	Mean (SD)	p-value	LBF
Time millisecond range (0.5 s)	Control	0.56 (0.1)	0.9	– 0.5
	DD	0.56 (0.1)		
Time second range (1.5 s)	Control	1.6 (0.3)	0.1	– 0.07
	DD	1.5 (0.3)

## 4 Discussion

In the current study we found that auditory time perception was strongly impaired in children with DD. Specifically, their sensory precision thresholds measured with a simple categorization task were about twice compared to age and non-verbal IQ matched peers without DD. The time perception impairment affected both the sub- and supra- second duration ranges.

The current results are consistent with previous studies reporting time perception impairments in individuals with DD either in the sub- (Pellerone, 2013, Vicario et al., 2012) or supra-second (Gilaie-Dotan et al., 2014, Hurks and Van Loosbroek, 2014, Skagerlund and Träff, 2014, Cester et al., 2017) duration ranges using visual stimuli, and extend it to the auditory domain.

The finding of time perception impairments in a disorder that hampers numerical processing has been previously interpreted as evidence in support of the ATOM theory (Skagerlund and Träff, 2014, Vicario et al., 2012). This theory proposes that numerical quantities are processed by a general system which also processes other continuous magnitudes, such as space and time, hosted in the parietal cortex (Walsh, 2003). If this system is impaired, as it can be hypothesized given the evidence for numerical impairments and for functional and anatomical abnormalities of the parietal cortex in individuals with DD, then it can be plausibly expected that other magnitudes are also affected. In this light, the current findings, showing that difficulties in individuals with DD span beyond the numerical domain and affect time perception, are in line with the ATOM theory. However, evidence in both neurotypicals and clinical populations challenged the original version of this theory. For example, studies in neurotypical individuals showed that the perceptual interaction across numerosity and time was not bidirectional (as it should be in the case of a single shared mechanism), with numerosity affecting duration processing but not vice versa (Cappelletti et al., 2009, Dormal and Pesenti, 2013) and suggested that the representational mechanisms for time and numerical processing might be partially independent. While methodological differences including the nature of the stimuli (simultaneous vs dynamic, Togoli et al., 2021) and/or the duration range tested can account for these differences, there is evidence for selective impairments of only one dimension following cortical lesion (Cappelletti et al., 2009). Likewise, previous studies reported that while individuals with DD performed poorly in numerosity tasks, their performance was comparable to neurotypical individuals when discriminating average item size (Castaldi et al., 2018), single object size (Anobile et al., 2018), cumulative area (Iuculano et al., 2008) and line length (Cappelletti et al., 2014, De Visscher et al., 2017). The relation between magnitude processing and mathematical abilities also does not generalize to all magnitudes. Precision of numerosity but not of size or density perception was found to be related to the normal development of mathematical abilities in children (Anobile et al., 2016) and education selectively improved sensitivity for numerosity, but not for single object size perception (Piazza et al., 2013). Overall, a more nuanced version of the ATOM theory conceiving the existence of partially shared and partially independent mechanisms to process distinct magnitudes, seems to fit better with these observations. While the current study was not designed to address this debate in the literature, as we tested only time processing, the results point to a clear impairment in a dimension other than number in DD, potentially supporting the existence of at least partially shared mechanisms.

According to the earliest and most influential theories of DD, this disorder was attributed to a core impairment either in the approximate number system dedicated to the approximate perception of large quantities (Dehaene, 1997), or in the object-tracking system dedicated to the exact processing of smaller sets of objects (Butterworth, 1999). However, findings of both cognitive and perceptual impairments that are not strictly confined to the numerical domain in individuals with DD underlined the multi-componential nature of this heterogeneous disorder (Fias et al., 2013, Fias, 2016, Iuculano, 2016, Castaldi et al., 2020). For example, impairments in various domain-general executive functions, such as working memory, attention, and cognitive control has been reported in individuals with DD, even when manipulating non-numerical material (Askenazi and Henik, 2010, Szűcs et al., 2013, Szűcs, 2016). Moreover, other studies found non-numerical impairments in individuals with DD even in very basic aspects of visual scene perception, such as lower symmetry and form perception sensitivity and enhanced visual crowding (Szűcs et al., 2013, Castaldi et al., 2020, Castaldi et al., 2022). The current results are the first reporting that impairments in individuals with DD can extend also to categorization of non-numerical auditory stimuli, further reinforcing a more comprehensive view of DD.

In the current study we tested time perception across two time ranges, sub- and supra- seconds. The rationale of testing these two ranges is that it has been proposed that time perception is not processed by a unitary mechanism, but rather that multiple “timers” operate over multiple time scales (Buhusi & Meck, 2005). These are often divided into three different ranges: circadian timing (important to represent day-night cycle to regulate sleep and metabolism), supra-second (or interval) timing and sub-second timing. Time processing at these different scales is thought to rely on partially distinct neural mechanisms (Buhusi & Meck, 2005). While it is well known that circadian timing is regulated by the activity of the suprachiasmatic nucleus of the hypothalamus, the neural substrate supporting supra- and sub-second timing is controversial. Meta-analyses of imaging studies found that a subcortical network important for motor control (including, but not limited to, basal ganglia and cerebellum) was more likely activated by perception of short intervals up to one second, whereas processing of longer supra-second intervals was supported by cortical areas, such as frontal and parietal cortices (Lewis and Miall, 2003b, Wiener et al., 2010). Reflecting the hierarchy of the neural networks on which they rely, processing of short and long durations has been considered as more automatic or more cognitively-mediated respectively. Along this line, previous studies have suggested that when judging longer intervals, adults might use cognitive strategies, such as counting even when they are told not to do so (Hinton & Rao, 2004). Accordingly, Gilaie-Dotan et al. (2014) proposed that the time perception impairments in the supra-second range observed in individuals with DD should be ascribed to their counting difficulty rather than to an impairment of their internal clock pacemaker or of a magnitude mechanism. We doubt this explanation can account for the current results: if counting strategies were used, we should have observed higher precision when categorizing supra-second compared to sub-second durations in the control group and the opposite in participants with DD, but this is not what we observed, as sensory thresholds were not different across duration ranges in either group. It is therefore unlikely that the supra-second time perception impairment observed in participants with DD in the current experiment can be secondary to a counting impairment. Rather these results are in line with a general impairment in the ability to process and categorize time.

Which can be the origin of such widespread time perception impairment in DD? As mentioned before, partially distinct neural networks have been identified for processing durations in the sub- and supra-second ranges. However, there is evidence of brain areas encoding both short (milliseconds) and longer (seconds) durations, suggesting that time processing is mediated by a distributed network that can be variably engaged depending on task requirements and time scale tested rather than by clearly segregated and dedicated networks (Wiener et al., 2010). For example, Lewis et al. (2003a) found that the bilateral insula, the dorsolateral prefrontal cortex, the right pre-supplementary motor area, the frontal pole and the inferior parietal cortex were activated during processing of both sub- and supra-second intervals. Importantly most of these regions (and many more) have shown both anatomical and functional impairments in individuals with DD. Moreover, anomalies have also been found in subcortical areas such as the cerebellum, putamen and many others (Iuculano, 2016) which have been more tightly related to sub-second time perception processing. Overall, the presence of both sub- and supra-second time perception impairments can be expected in light of the multiple cortical and subcortical abnormalities observed in individuals with DD.

The current study is not exempt from limitations. Because the finding of carefully screened children with DD available for participating in research studies is not easy, the sample size of the current and of all previous studies is relatively small. For this reason, despite the power analysis suggesting the acceptability of the sample size, we additionally reported Bayes factors to all the statistical comparisons supporting the most relevant claims. We found at least positive, but also strong evidence in support of time perception impairments in participants with DD compared to the control group. Moreover, despite the fact that we took particular care in ensuring that the age range variability was comparable between the DD and control groups, it spanned several years. Future studies should test time perception in larger groups pf participants and provide a finer characterization of the developmental changes of this ability in relation to math. Finally, reading abilities were not always reported in previous studies investigating time perception in dyscalculia and in the current experiment reading scores were not available for all participants so we could not covary them out. Since time perception impairments when judging both visual and auditory events have been reported also in children with developmental dyslexia (Tallal, 1980, Gooch et al., 2011, Khan et al., 2014, Plourde et al., 2017, Casini et al., 2018), which is often present in comorbidity with dyscalculia, future studies should test whether auditory time perception impairments can also be observed in profiles with pure dyscalculia or whether these are only present in comorbid dyslexia-dyscalculia profiles.

In sum, with the present study we report auditory time perception impairments in children with DD which impacts multiple time scales. This finding expands our understanding of DD and contributes to framing it as a complex disorder affecting various basic perceptual abilities spanning beyond the numerical domain. Future research should explore auditory perception in individuals with DD using tasks unrelated to magnitude processing, such as meter, rhythm, or auditory sequences perception. This could offer new avenues for rehabilitation, such as musical training which is known to enhance reading abilities plausibly by improving children timing skills (Moreno et al., 2009, Flaugnacco et al., 2015, Rolka and Silverman, 2015). Given the emerging evidence linking timing and math abilities, musical training may also have beneficial effects on math skills.

## CRediT authorship contribution statement

**Elisa Castaldi:** Conceptualization, Data curation, Formal analysis, Funding acquisition, Investigation, Methodology, Project administration, Resources, Software, Supervision, Validation, Visualization, Writing – original draft, Writing – review & editing. **Filippo Gasperini:** Conceptualization, Investigation, Writing – review & editing. **Francesca Tinelli:** Conceptualization, Investigation, Writing – review & editing. **Giovanni Anobile:** Conceptualization, Data curation, Formal analysis, Funding acquisition, Investigation, Methodology, Project administration, Resources, Software, Supervision, Validation, Visualization, Writing – original draft, Writing – review & editing. **Mariaelisa Bartoli:** Conceptualization, Investigation, Writing – review & editing.

## Declaration of Competing Interest

None.

## Data Availability

The results from this study are publicly available at: https://zenodo.org/records/10966052
